# A new representation method of the relative position between objects in the image based on the histogram of position sensing forces

**DOI:** 10.1038/s41598-024-51396-x

**Published:** 2024-01-08

**Authors:** Zeyu Tian

**Affiliations:** 1 College of Surveying and Mapping Engineering, Heilongjiang Institute of Technology, Harbin, 150050 China; 2 Xi’an Research Institute of Surveying and Mapping, Xi’an, 710054 China; 3 State Key Laboratory of Geo-Information Engineering, Xi’an, 710054 China

**Keywords:** Computer science, Information technology

## Abstract

Let the computer apprehend and describe the representation of the relative position between objects of the image by the way of the common intuition of the human is an important task of the computer vision and pattern recognition. To complete this task, the position sensing parameter and histogram of position sensing forces are proposed in this paper. The position sensing parameter can represent the spatial relative position of the point with regard to the reference object, and the point is from the argument object. The histogram of position sensing forces is composed of the position sensing parameter of each point in the argument object and the gravitational forces between each point of the argument object and each point of the reference object. The histogram of position sensing forces can simulate the human perception for the directional spatial relations between the argument object and reference object of the image, considering the shape, size, angular and metric information of the spatial object.

## Introduction

Let the computer apprehend and describe the representation of the relative position between objects of the image by the way of the human common space perception, which is an important task of the computer vision and pattern recognition. This task which is useful to understand the scenes of the image and bridge the gap between the pixels and semantics of the image, can promote the development of applications such as the linguistic description of spatial relations^1^, object recognition, scene recognition, scene matching^2^, content-based image retrieval^3,4^, robot navigation, etc. But most representation approaches of the relative position between objects in the image are not a good simulation of the common intuition of the human.

Several formal methods of assessing the relative position between objects have been proposed in the literatures. These methods are mainly divided into two categories. The first kind of methods usually abstract a spatial object as a point or fits the spatial object to mini-mum bounding rectangle (MBR) or Voronoi polygon. The first kind of methods mainly includes the cone-shaped directions model^5^, projection-based model^6^, and direction relation matrix model^7,8^, etc. The first kind of methods ignores the influence of the size and shape of the spatial object and cannot provide the accurate description of the directional spatial relations. Different from the first kind of methods, the second kind of methods doesn’t abstract or fit the spatial object, which can consider the size and shape of the spatial object and provide more accurate description of the directional spatial relations than the first kind of methods. The second kind of methods includes the histogram of angles^9^, the R-histogram^10^, R*-histogram^11^, Quad-tree histogram^12^, etc. The analysis of the second kind of methods are as follows.

The histogram of angles^9^ is introduced to define the fuzzy relative spatial position. The histogram of angles is computed from the frequency of the angles, and the angles are between any two points in the argument object and reference object. The computational complexity of the histogram of angles is high and metric information is not taken into account. Based on the histogram of angles, with the introduction of the labeled distance, the R-histogram^10^ is proposed to describe the directional and topological spatial relations. R-histogram only calculates the pairs of points on the boundaries of the argument object and reference object, which improves the computational efficiency. But the description of the directional spatial relation of R-histogram is poor, and the description of the topological relation of R-histogram is also not accurate. R*-histogram^11^ considers more pairs of points than R-histogram. Quad-tree histogram^12^ divides the objects into a number of regular sub-objects and calculates angle-histogram of centroid points of sub-objects. Quad-tree histogram ignores some shape information of the object, but greatly improves the computational efficiency.

In^13^ and^14^, the histogram of forces is proposed to assess the directional spatial relations between areal objects. According to different parameters, the histogram of forces consists of F0-histogram (histogram of constant forces) and F2-histogram (histogram of gravitational forces). The F0-histogram is the sum of weights between longitudinal sections of the argument object and reference object. The F0-histogram can provide a global view and consider the closest parts and the farthest parts of the objects equally. But the F0-histogram cannot take into account the metric information. The F2-histogram is computed from the scalar resultant of gravitational forces between longitudinal sections of the argument object and reference object. But F2-histogram only focuses on the closest parts of the objects and cannot provide a global view. F0-histogram and F2-histogram have both advantages and disadvantages. In^1^, a new method of the handling of histograms is proposed. This method can combine the F0-histogram’s opinion with the F2-histogram’s opinion and define the new fuzzy directional relations. Although this method can give consideration to the F0-histogram’s opinion and F2-histogram’s opinion and produce logical linguistic descriptions, this method does not improve the histogram of forces and still relies on the computation of the histogram of forces in^13^ and^14^. In^15^, the computational complexity of the histogram of forces is reduced to $O\left( {N\log N} \right)$. In^16^, the histogram of forces is used to describe the special directional relation such as ‘among’, ‘between’ and ‘surround’. In^17^, the concepts of spatial template and F-template are proposed to identify which object that best satisfies a given relationship to a reference object. In^18^ and^19^, the histogram of forces are coupled with Allen’s relationships to extract the fuzzy topological between different objects. In^20^, the histogram of forces is extended to handle 3D vector objects. In^3^ and^21^, the histogram of forces reacts well to affine transformations and the similarity of spatial configureurations is evaluated by the histograms of forces. In^22^ and^23^, the φ-descriptor is an extension of the histogram of forces using 13 different histograms and provides a generic framework to assess the usual spatial relations. In^24^, a stack of force histograms using various force parameters constitutes the descriptor “(discrete) Force Banner” (dFB), which is translated into the spatial relations in the natural language by the machine learning.

In^25,26^, a morphological approach is proposed. In this approach, the argument object is compared to the fuzzy landscape attached to the reference object. This approach can provide an evaluation as two extreme values and an average value. In^27^, a fuzzy landscape model is proposed, which allows to visualize and evaluate this relation directly in the image space.

Among the various relative position descriptors, the histogram of forces is widely used. But the evaluations of the directional spatial relations by the histogram of forces are not accurate enough, and are sometimes different from the common intuition of the human. In this paper, based on the histogram of forces, we introduce the position sensing parameter and present the histogram of position sensing forces to simulate the common intuition of the human for the directional spatial relations.

## Methods

**Figure 1:**
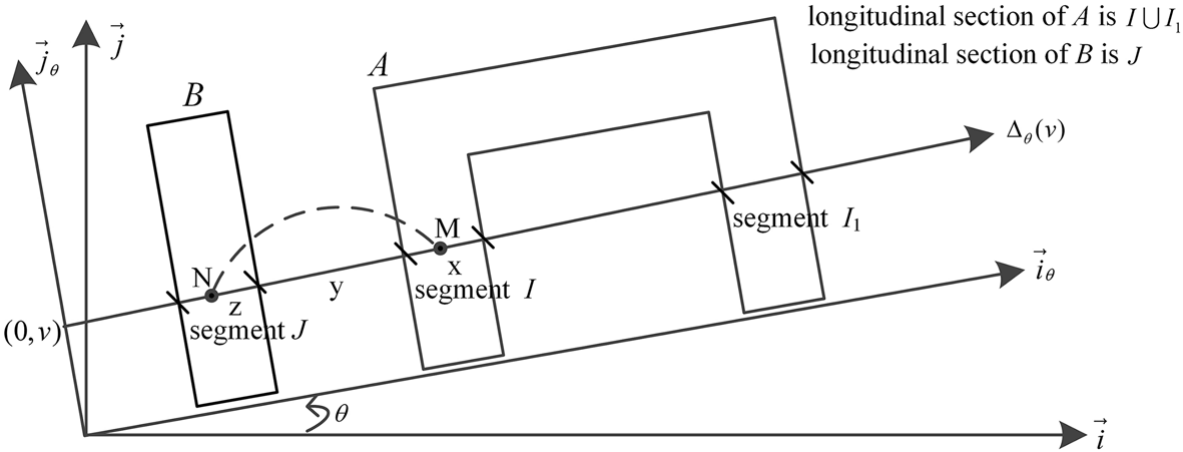
Frame $\left( {O,\overrightarrow {i}_{\theta } ,\overrightarrow {j}_{\theta } } \right)$, oriented straight line and longitudinal section.

The Euclidean plane is denoted by S where the argument object and reference object are defined, and the relative position of argument objects and reference object can be represented by the fuzzy directional spatial relation. As shown in Fig. 1, the plane S is referred to as a directional orthogonal frame $\left( {O,\overrightarrow {i} ,\overrightarrow {j} } \right)$. Assuming the angle $\theta$ represents the argument object *A* in the direction $\theta$ of the reference object *B*, the range of angle $\theta$ is [0,360) degrees and angle $\theta$ is real. And the frame $\left( {O,\overrightarrow {i}_{\theta } ,\overrightarrow {j}_{\theta } } \right)$ is obtained from the $\theta$ angle rotation of the frame $\left( {O,\overrightarrow {i} ,\overrightarrow {j} } \right)$ in Fig. 1. Within the frame $\left( {O,\overrightarrow {i}_{\theta } ,\overrightarrow {j}_{\theta } } \right)$, the oriented line $\Delta_{\theta } (v)$ is defined by the vector $\overrightarrow {i}_{\theta }$ and the point of coordinates $\left( {0,v} \right)$.

If object *A* and *B* are points, the definition of the directional spatial relation is explicit in^7^. If objects *A* and *B* are areal objects, because the shape, size, orientation, and distance are involved, the directional spatial relation is complex.

**Figure 2:**
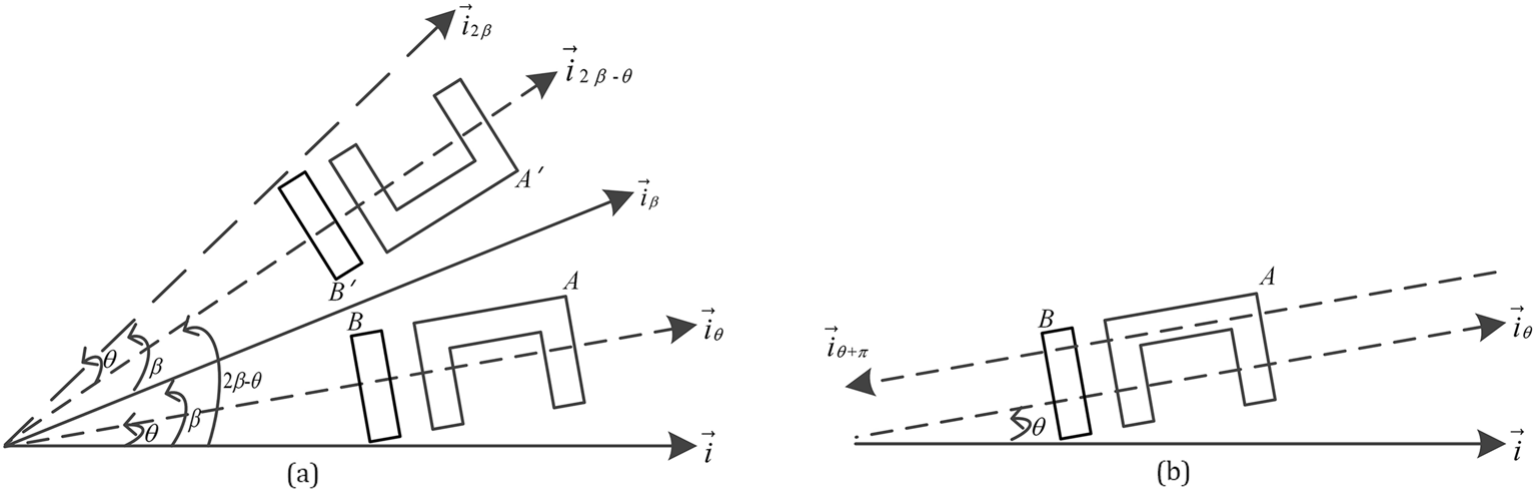
Basic properties [P3] and [P4].

In order to represent the directional spatial relation between areal objects, the histogram of forces^13^ computes the gravitational forces between each point of the argument object and each point of the reference object. In this paper, the histogram of position sensing forces computes the spatial position sensing parameters and gravitational forces between each point of the argument object and each point of the reference object. And the histogram of position sensing forces has the four basic properties:

[P1] two objects can be assimilated to points if they are distant enough; [P2] the directional relations are not sensitive to scale changes; [P3] Let object *A* and *B* be orthogonal symmetry with respect to $\overrightarrow {i}_{\beta }$-axis to obtain object *A’* and *B’*, $\beta$ is the angle between the vector $\overrightarrow {i}_{\beta }$ and horizontal vector $\overrightarrow {i}$, if the object *A* is in the direction $\theta$ of object *B,* and object *A’* is in the direction $2\beta { - }\theta$ of object *B’* (Fig. 2a); [P4] The object *A* is in the direction $\theta$ of object *B* and object *B* is in the direction $\theta + \pi$ of object *A* (Fig. 2b).

## Spatial position sensing

**Figure 3:**
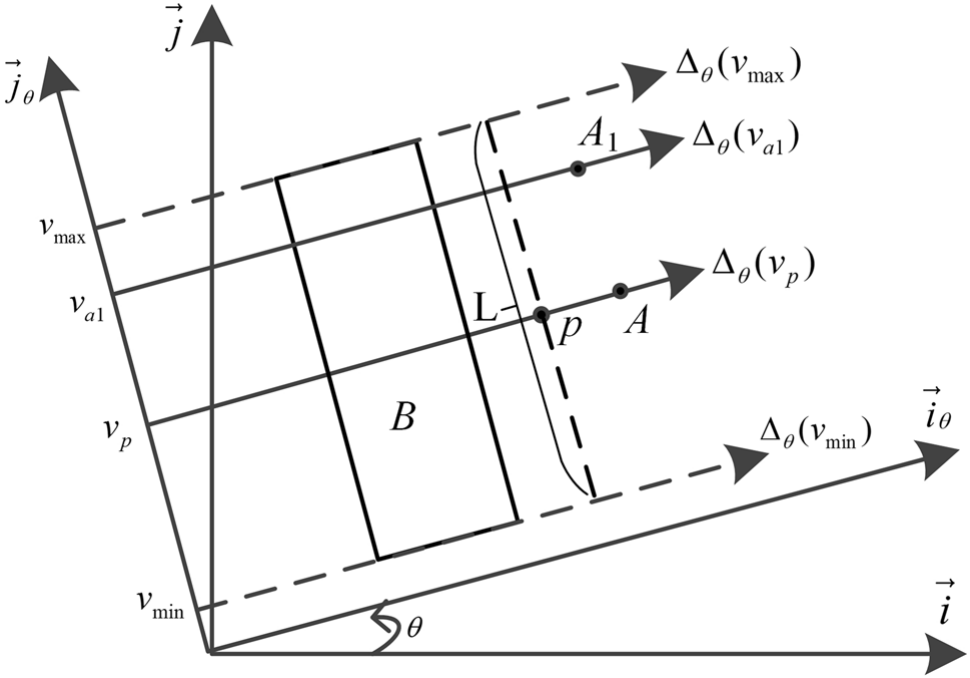
Exact direction and inexact direction.

The spatial position sensing parameter of the histogram of position sensing forces represents the relative position of the point with regard to the reference areal object. Let objects *A* and *A*_1_ be the points, and object *B* be the areal object. As shown in Fig. 3, *A* is in the exact direction $\theta$ of *B* and *A*_1_ is in the inexact direction $\theta$ of *B.*

The exact direction $\theta$ and inexact direction $\theta$ are specified as follows:

### Definition 1

Within the frame $\left( {O,\overrightarrow {i}_{\theta } ,\overrightarrow {j}_{\theta } } \right)$, the object *A* is the point, the object *B* is the areal object, $v_{\max }$ and $v_{\min }$ are maximum and minimum coordinate values of the areal object *B* projected to the coordinate axis $\overrightarrow {j}_{\theta }$, the oriented lines $\Delta_{\theta } (v_{\max } )$ and $\Delta_{\theta } (v_{\min } )$ are defined by the vector $\overrightarrow {i}_{\theta }$ and points $\left( {0,v_{\max } } \right)$ and $\left( {0,v_{\min } } \right)$, line segment *L* is perpendicular to the oriented lines $\Delta_{\theta } (v_{\max } )$ and $\Delta_{\theta } (v_{\min } )$, The midpoint of segment *L* is point *p*, and the oriented line $\Delta_{\theta } (v_{p} )$ is through point *p*. If point* A* is on the oriented line $\Delta_{\theta } (v_{p} )$, point* A* is in the exact direction $\theta$ of object* B*. If point* A* is not on the oriented line $\Delta_{\theta } (v_{p} )$, point* A* is in the inexact direction $\theta$ of object* B*, such as *A*_1_, point* A*_1_ is on the oriented line $\Delta_{\theta } (v_{a1} )$.

The exact direction $\theta$ and inexact direction $\theta$ both support the proposition “point *A* is in the direction $\theta$ of object *B*”. But the support degrees are different. If point *A* is in the exact direction $\theta$ of object *B*, the degree of truth of the proposition “point *A* is in the direction $\theta$ of object *B*” is the maximum. If point *A* is in the inexact direction $\theta$ of object *B*, the degree of truth of the proposition “point *A* is in the direction $\theta$ of object *B*” is involved with the shape and size of the reference object *B*.

If argument object *A* and reference object *B* both are points, because reference object *B* is point without the shape and size of the areal object, point *A* is in the exact direction $\theta$ of point *B*, and there is no such situation that point *A* is in the inexact direction $\theta$ of point* B*.

The position sensing parameter $\lambda$ can quantitatively describe the relative position of the argument point with regard to the reference object in the exact direction and inexact direction.

If the argument point *A* is in the direction $\theta$ of the reference object *B* and the relative position of *A* with regard to *B* changes, the degree of truth of the proposition “point* A* is in the direction $\theta$ of object *B*” will change simultaneously. In the direction $\theta$ of object *B*, the relative position of point *A* with regard to object *B* is represented by the position sensing parameter $\lambda$. If point* A* is in the exact direction $\theta$ of object* B*, the position sensing parameter $\lambda$ is equal to 1.0. If point* A* is in the inexact direction $\theta$ of object* B*, the position sensing parameter $\lambda$ is less than 1.0. The relative position of point *A* with regard to object *B* is represented by the position sensing parameter $\lambda$,$\lambda$ is specified as follows:

### Definition 2

$l_{above} (\theta ,v_{a} )$ denotes the distance between the oriented line $\Delta_{\theta } (v_{a} )$ and $\Delta_{\theta } (v_{\max } )$,$l_{below} (\theta ,v_{a} )$ denotes the distance between the oriented line $\Delta_{\theta } (v_{a} )$ and $\Delta_{\theta } (v_{\min } )$. Let $l_{above} (\theta ,v_{a} )$ and $l_{below} (\theta ,v_{a} )$ be the function from ${\mathbb{R}} \times {\mathbb{R}}$ into ${\mathbb{R}}_{ + }$,$\theta$ and $\nu$ be two reals. The function $\lambda$ from ${\mathbb{R}} \times {\mathbb{R}}$ into [0,1] defined by the following formula:

1 $$\lambda (\theta ,\nu ) = \left\{ \begin{gathered} l_{above} (\theta ,\nu )/l_{below} (\theta ,\nu ) \, v_{p} \le v_{a} \hfill \\ l_{below} (\theta ,\nu )/l_{above} (\theta ,\nu ) \, v_{p} > v_{a} \hfill \\ \end{gathered} \right.$$

And if object *A* and *B* both are points, point *A* is in the exact direction $\theta$ of point* B*, and $\lambda (\theta ,\nu ) = 1.0$.

**Figure 4:**
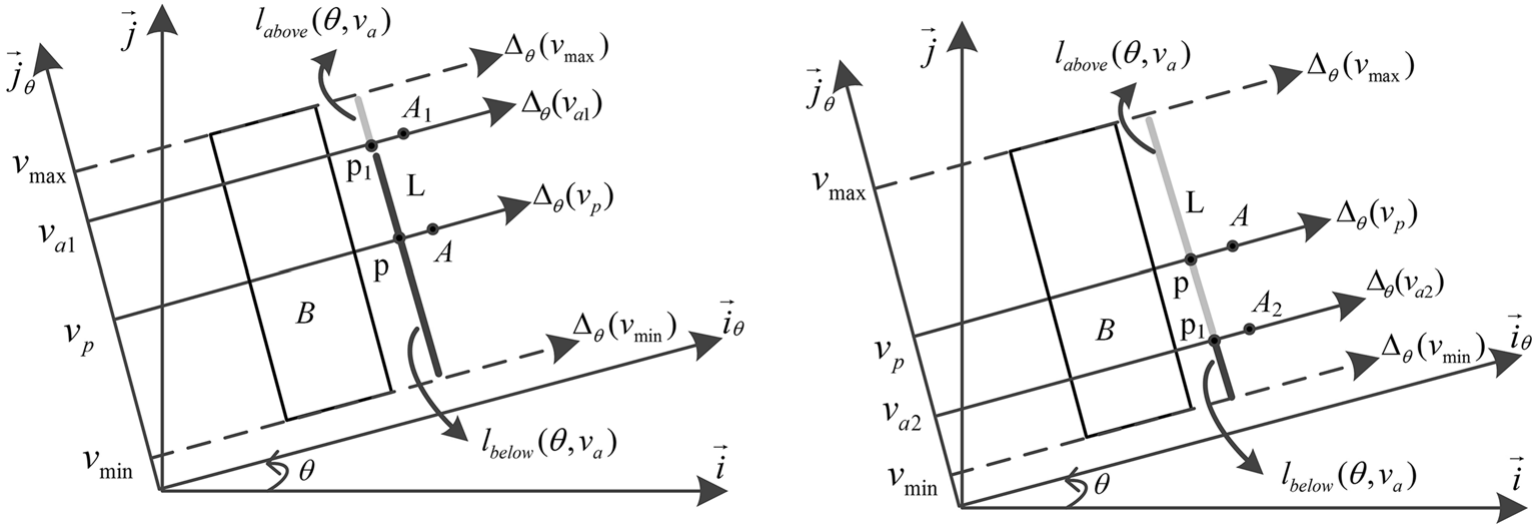
Position sensing parameter $\lambda$.

In Fig. 4, the coordinates $v_{p} = v_{a}$, the oriented line $\Delta_{\theta } (v_{p} )$ and $\Delta_{\theta } (v_{a} )$ coincide, point* A* is in the exact direction $\theta$ of object* B*,$l_{above} (\theta ,v_{a} ) = l_{below} (\theta ,v_{a} )$, and the position sensing parameter $\lambda = 1.0$. The coordinates $v_{p} < v_{a1}$, point* A*_1_ is in the inexact direction $\theta$ of object* B*, and the position sensing parameter $\lambda = l_{above} /l_{below}$. The coordinates $v_{p} > v_{a2}$, point* A*_2_ is in the inexact direction $\theta$ of object* B*, and the position sensing parameter $\lambda = l_{below} /l_{above}$.

The position sensing parameter $\lambda$ represents the spatial relative position of the argument point with regard to the reference object, considering both shape and size information of the reference areal object.

According to Definition 2, the position sensing parameter is computed by the ratio of the distance, which is unchanged for scale changes and orthogonal symmetry of the argument and reference objects. And if object *A* and *B* both are points, the position sensing parameter is 1.0. So the position sensing parameter satisfies properties [P1], [P2] and [P3].

## Histogram of position sensing forces

Based on the concept of the spatial position sensing and position sensing parameter $\lambda$, the histogram of position sensing forces can represent the relative position of the argument object with regard to the reference object. The position sensing parameter $\lambda$ can represent the relative position of the argument point with regard to the reference object in the exact direction and inexact direction.

The histogram of position sensing forces computes the position sensing parameter $\lambda$ of the point with regard to the reference object, the point is from the argument object, and simultaneously computes the gravitational forces between each point of the argument object and each point of the reference object.

Within the frame $\left( {O,\overrightarrow {i}_{\theta } ,\overrightarrow {j}_{\theta } } \right)$, the object *A* is the argument, the object *B* is the reference,$\Delta_{\theta } (v)$ is the oriented line, and let $\theta$ and $\nu$ be two reals. If object *A* and oriented line $\Delta_{\theta } (v)$ intersect,$A \cap \Delta_{\theta } (v)$ is the union of a finite number of mutual disjoint segments. If object *B* and oriented line $\Delta_{\theta } (v)$ intersect,$B \cap \Delta_{\theta } (v)$ is the union of a finite number of mutual disjoint segments. The longitudinal section of object *A* on the direction $\theta$ is denoted by $A_{\theta } (v){ = }A \cap \Delta_{\theta } (v)$. The longitudinal section of object *B* on the direction $\theta$ is denoted by $B_{\theta } (v){ = }B \cap \Delta_{\theta } (v)$. As shown in the Fig. 1, the oriented line $\Delta_{\theta } (v)$ and object *A* intersect at two segments $I$ and $I_{1}$, the oriented line $\Delta_{\theta } (v)$ and object *B* intersect at a segment $J$, the longitudinal section of *A* is $A_{\theta } (v) = I \cup I_{1}$, and the longitudinal section of *B* is $B_{\theta } (v) = J$. And T is denoted by the set of triples $\left( {\theta ,A_{\theta } (v),B_{\theta } (v)} \right)$.

The histogram of position sensing forces is computed by the function $\varphi$-for the handling of points, the function $f_{\theta }^{\nu }$*–*for the handling of segments, and the function *F-*for the handling of longitudinal sections.

## Function $\varphi$ for the handling of points

Let *M* be a point of the argument object *A*, *N* be a point of the reference object *B*, *M* and *N* be on the oriented line $\Delta_{\theta } (v)$ (Fig. 1). The relative position of *M* and *N* on the oriented line $\Delta_{\theta } (v)$ is represented by the function $\varphi$ from ${\mathbb{R}}$ into ${\mathbb{R}}_{ + }$. The function $\varphi$ is defined by:

2 $$\varphi (m - n) = \frac{1}{{(m - n)^{2} \, }} \, m > n$$

$m$ and $n$ refer to the respective abscissas of points *M* and *N* on $\Delta_{\theta } (v)$. If $m > n$, *M* is in the direction $\theta$ of *N*. If $m \le n$,$\varphi (m - n)$ is null, *M* is not in the direction $\theta$ of *N*.

The $\left( {M,N} \right)$ couple is considered as an argument to support the proposition “*A* is in the direction $\theta$ of *B*”. The value $\varphi (m - n)$ is the weight of this argument. The weight $\varphi (m - n)$ is interpreted as the gravitational force. According to Definition 2, the position sensing parameter $\lambda$ of the point* M* with regard to the reference object *B* is $\lambda (\theta ,\nu )$.

## Function $f_{\theta }^{\nu }$ for the handling of segments

**Figure 5:**
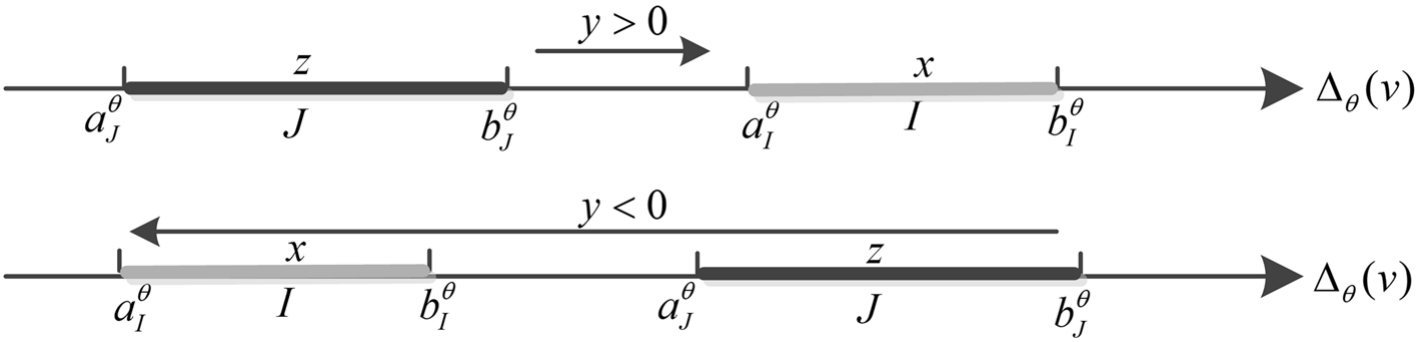
Relative position of segments *I* and* J*.

Let *I* be a segment of the set $A_{\theta } (v)$, *J* be a segment of the set $B_{\theta } (v)$, *M* be a point of segment *I*, and *N* be a point of segment *J* (Fig. 1). The respective abscissas of the ends of segments *I* and* J* are noted $a_{I}^{\theta }$,$b_{I}^{\theta }$,$a_{J}^{\theta }$ and $b_{J}^{\theta }$ on $\Delta_{\theta } (v)$, with $a_{I}^{\theta }$ lower than $b_{I}^{\theta }$, and $a_{J}^{\theta }$ lower than $b_{J}^{\theta }$ (Fig. 5). The lengths of segments *I* and* J* are denoted by $x = b_{I}^{\theta } - a_{I}^{\theta }$ and $z = b_{J}^{\theta } - a_{J}^{\theta }$. The difference $a_{I}^{\theta } - b_{J}^{\theta }$ is denoted by *y*. The relative position of segments* I* and *J* on the oriented line $\Delta_{\theta } (v)$ is represented by the function $f$ from ${\mathbb{R}}_{ + } \times {\mathbb{R}} \times {\mathbb{R}}_{ + }$ into ${\mathbb{R}}_{ + }$. The function $f$ is defined by the following formula:

3 $$f(x,y,z) = \int_{{a_{I}^{\theta } }}^{{b_{I}^{\theta } }} {\left( {\int_{{b_{J}^{\theta } }}^{{a_{J}^{\theta } }} {\varphi (m - n)dn} } \right)dm}$$

$f(x,y,z)$ is computed by summing the weights $\varphi (m - n)$ of the $\left( {M,N} \right)$ arguments.

If $y > 0$, segment *I* is in the direction $\theta$ of segment* J*, the formula (3) is represented as follows:

4 $$\begin{aligned} f(x,y,z) & = \int_{{a_{I}^{\theta } }}^{{b_{I}^{\theta } }} {\left( {\int_{{b_{J}^{\theta } }}^{{a_{J}^{\theta } }} {\varphi (m - n)dn} } \right)dm} \\ & { = }\int_{{a_{I}^{\theta } }}^{{b_{I}^{\theta } }} {\left( {\int_{{b_{J}^{\theta } }}^{{a_{J}^{\theta } }} {\frac{1}{{(m - n)^{2} \, }}dn} } \right)dm} \\ & { = }\ln \frac{{(b_{I}^{\theta } - b_{J}^{\theta } )(a_{I}^{\theta } - a_{J}^{\theta } )}}{{(a_{I}^{\theta } - b_{J}^{\theta } )(b_{I}^{\theta } - a_{J}^{\theta } )}} \\ \end{aligned}$$

If $x + y + z \le 0$, $f(x,y,z) = 0$ and segment *I* is not in the direction $\theta$ of segment* J*.

The function $f(x,y,z)$ only represents the relative position of segments* I* and *J* on the oriented line $\Delta_{\theta } (v)$, cannot represent the relative position of segment* I* with regard to the reference object *B*. So the position sensing parameter $\lambda$ is introduced to $f(x,y,z)$. According to Definition 2, the position sensing parameter $\lambda$ of point *M* with regard to the reference object *B* is $\lambda (\theta ,\nu )$, and point *M* is any point of segment *I*. The relative position of segments* I* and *J* on the oriented line $\Delta_{\theta } (v)$ and relative position of segment* I* with regard to the reference object *B* are represented by the Definition 3 of the function $f_{\theta }^{\nu }$.

### Definition 3

The function $f_{\theta }^{\nu }$ from ${\mathbb{R}}_{ + } \times {\mathbb{R}} \times {\mathbb{R}}_{ + }$ into ${\mathbb{R}}_{ + }$ defined by the following formula is called the function generated by $f$ and $\lambda (\theta ,\nu )$:

5 $$\begin{gathered} f_{\theta }^{\nu } (x,y,z) = \int_{{a_{I}^{\theta } }}^{{b_{I}^{\theta } }} {\left( {\int_{{b_{J}^{\theta } }}^{{a_{J}^{\theta } }} {\lambda (\theta ,\nu ) \times \varphi (m - n)dn} } \right)dm} \\ = \lambda (\theta ,\nu ) \times \int_{{a_{I}^{\theta } }}^{{b_{I}^{\theta } }} {\left( {\int_{{b_{J}^{\theta } }}^{{a_{J}^{\theta } }} {\varphi (m - n)dn} } \right)dm} \\ = \lambda (\theta ,\nu ) \times f(x,y,z) \\ \end{gathered}$$

If $y > 0$, segment *I* is in the direction $\theta$ of segment* J*. If $x + y + z \le 0$,$f_{\theta }^{\nu } (x,y,z) = 0$ and segment *I* is not in the direction $\theta$ of segment* J*. The above are shown in Fig. 4. The $\left( {I,J} \right)$ couple is considered as an argument to support the proposition “*A* is in the direction $\theta$ of *B*”. The value $f_{\theta }^{\nu } (x,y,z)$ is the weight of this argument.

## Function *F* for the handling of longitudinal sections

The longitudinal section $A_{\theta } (v)$ includes *n* segments and $A_{\theta } (v) = \bigcup\nolimits_{i = 1 \cdots n} {I_{i} }$. The longitudinal section $B_{\theta } (v)$ includes *m* segments and $B_{\theta } (v) = \bigcup\nolimits_{j = 1 \cdots m} {J_{j} }$. The relative position of longitudinal sections $A_{\theta } (v)$ and $B_{\theta } (v)$ on the oriented line $\Delta_{\theta } (v)$ is represented by the function *F* from $T = \left( {\theta ,A_{\theta } (v),B_{\theta } (v)} \right)$ into ${\mathbb{R}}_{ + }$. The function *F* is defined by the following formula:

6 $$F\left( {\theta ,A_{\theta } (v),B_{\theta } (v)} \right) = \sum\limits_{i = 1 \cdots n,j = 1 \cdots m} {f_{\theta }^{\nu } (x_{i} ,y_{ij} ,z_{j} )}$$

$F\left( {\theta ,A_{\theta } (v),B_{\theta } (v)} \right)$ is computed by summing the weights $f_{\theta }^{\nu } (x,y,z)$ of the $\left( {I,J} \right)$ arguments.

The $\left( {A_{\theta } (v),B_{\theta } (v)} \right)$ couple is considered as an argument to support the proposition “*A* is in the direction $\theta$ of *B*”. The value $F\left( {\theta ,A_{\theta } (v),B_{\theta } (v)} \right)$ is the weight of this argument.

## Histogram of position sensing forces

The relative position of object *A* with regard to object *B* is represented by the function $F_{P}^{AB}$ from ${\mathbb{R}}$ into ${\mathbb{R}}_{ + }$. The function $F_{P}^{AB}$ is defined by the following formula:

7 $$F_{P}^{AB} \left( \theta \right) = \int_{ - \infty }^{ + \infty } {F\left( {\theta ,A_{\theta } (v),B_{\theta } (v)} \right)dv}$$

$F_{P}^{AB} \left( \theta \right)$ is the total weight of the arguments in favor of the proposition “*A* is in the direction $\theta$ of *B*” and represents the scalar resultant of elementary gravitational forces with position sensing parameters.

When any numbers of values are taken from [0,360) degrees to form a serie of angles $\theta$, for example, each degree within the range [0,360) degrees is taken a value to form a serie of angles $\theta$, and a serie of the function $F_{P}^{AB} \left( \theta \right)$ is obtained through formula (7). The serie of the function $F_{P}^{AB} \left( \theta \right)$ is called the histogram of position sensing forces associated with (*A, B*) via $F_{P}^{AB}$, and is also called $F_{P}$-histogram associated with (*A, B*).

## Handling of the histogram of position sensing forces

The histogram $F_{P}^{AB}$ of position sensing forces obtained through formula (7) from a serie of angles $\theta$ within [0,360) degrees is a set of the scalar resultants of elementary gravitational forces with position sensing parameters.

The relative position of objects *A* and *B* can be represented by the fuzzy directional spatial relation $\Re_{\alpha }$.$\Re_{\alpha }$ connects objects *A* and *B* with an element of interval [0,1]. The proposition $A\Re_{\alpha } B$ is stated as “*A* is in the direction $\alpha$ of *B*”,$\alpha$ is an angle within [0,360) degrees.$\Re_{\alpha } (A,B)$ is the degree of truth of the proposition $A\Re_{\alpha } B$.

To obtain the degree $\Re_{\alpha } (A,B)$ of truth, the map $H_{u}^{\alpha }$^13^ is introduced to handle the histogram $F_{P}^{AB}$, and $\Re_{\alpha } (A,B)$ is defined by the following formula:

8 $$\begin{aligned} \Re_{\alpha } (A,B) & = H_{u}^{\alpha } (F_{P}^{AB} (\theta )) \\ & { = }sum(F_{P}^{AB} (\theta ) \times \mu (\theta ))/sum(F_{P}^{AB} (\theta )) \, \theta \in [\alpha - {\pi \mathord{\left/ {\vphantom {\pi 2}} \right. \kern-0pt} 2},\alpha + {\pi \mathord{\left/ {\vphantom {\pi 2}} \right. \kern-0pt} 2}] \\ \end{aligned}$$

Within the angle range $\theta \in [\alpha - {\pi \mathord{\left/ {\vphantom {\pi 2}} \right. \kern-0pt} 2},\alpha + {\pi \mathord{\left/ {\vphantom {\pi 2}} \right. \kern-0pt} 2}]$, the handling of the histogram $F_{P}^{AB}$ needs to calculate the sum of $F_{P}^{AB} (\theta ) \times \mu (\theta )$ divided by the sum of $F_{P}^{AB} (\theta )$. And $\mu (\theta )$ is the membership function.

Because $\Re_{\alpha } (A,B)$ is the degree of truth of the proposition “*A* is in the direction $\alpha$ of *B*”, these values of the histogram $F_{P}^{AB}$ within the angle range $\theta \in [\alpha - {\pi \mathord{\left/ {\vphantom {\pi 2}} \right. \kern-0pt} 2},\alpha + {\pi \mathord{\left/ {\vphantom {\pi 2}} \right. \kern-0pt} 2}]$ all support this proposition, but these values have different support degrees for this proposition. The value of the histogram $F_{P}^{AB}$ at the angle $\alpha$ has the highest support degree for this proposition, other values of the histogram $F_{P}^{AB}$ within the angle range $\theta \in [\alpha - {\pi \mathord{\left/ {\vphantom {\pi 2}} \right. \kern-0pt} 2},\alpha + {\pi \mathord{\left/ {\vphantom {\pi 2}} \right. \kern-0pt} 2}]$ also support this proposition to a certain extent. The histogram $F_{P}^{AB}$ that is not within the angle range $\theta \in [\alpha - {\pi \mathord{\left/ {\vphantom {\pi 2}} \right. \kern-0pt} 2},\alpha + {\pi \mathord{\left/ {\vphantom {\pi 2}} \right. \kern-0pt} 2}]$ definitely does not support this proposition. So only the histogram $F_{P}^{AB}$ within the angle range $\theta \in [\alpha - {\pi \mathord{\left/ {\vphantom {\pi 2}} \right. \kern-0pt} 2},\alpha + {\pi \mathord{\left/ {\vphantom {\pi 2}} \right. \kern-0pt} 2}]$ needs to be considered.

Taking the calculation of the degree of truth of the proposition “the argument object *A* to the right, left, above and below of the reference object *B*” as an example, “the object *A* to the right of the object *B*” is “*A* is in the direction $\alpha { = }0$ of *B*”, “the object *A* to the left of the object *B*” is “*A* is in the direction $\alpha { = }\pi$ of *B*”, “the object *A* to the above of the object *B*” is “*A* is in the direction $\alpha { = }{\pi \mathord{\left/ {\vphantom {\pi 2}} \right. \kern-0pt} 2}$ of *B*”, “the object *A* to the below of the object *B*” is “*A* is in the direction $\alpha { = - }{\pi \mathord{\left/ {\vphantom {\pi 2}} \right. \kern-0pt} 2}$ of *B*”, and the formula (8) of $\Re_{\alpha } (A,B)$ is expressed as:

9 $$\Re_{\alpha } (A,B) = \left\{ {\begin{array}{*{20}l} {sum(F_{P}^{AB} (\theta ) \times \mu_{right} (\theta ))/sum(F_{P}^{AB} (\theta ))} \hfill & {\quad {{ - \pi } \mathord{\left/ {\vphantom {{ - \pi } 2}} \right. \kern-0pt} 2} \le \theta \le {\pi \mathord{\left/ {\vphantom {\pi 2}} \right. \kern-0pt} 2}} \hfill \\ {sum(F_{P}^{AB} (\theta ) \times \mu_{left} (\theta ))/sum(F_{P}^{AB} (\theta ))} \hfill & {\quad \pi \le \theta \le {{ - \pi } \mathord{\left/ {\vphantom {{ - \pi } 2}} \right. \kern-0pt} 2}, \, {\pi \mathord{\left/ {\vphantom {\pi 2}} \right. \kern-0pt} 2} \le \theta \le \pi } \hfill \\ {sum(F_{P}^{AB} (\theta ) \times \mu_{above} (\theta ))/sum(F_{P}^{AB} (\theta ))} \hfill & {\quad {0} \le \theta \le \pi } \hfill \\ {sum(F_{P}^{AB} (\theta ) \times \mu_{below} (\theta ))/sum(F_{P}^{AB} (\theta ))} \hfill & {\quad - \pi \le \theta \le 0} \hfill \\ \end{array} } \right.$$

$\mu_{right} (\theta )$, $\mu_{left} (\theta )$, $\mu_{above} (\theta )$ and $\mu_{below} (\theta )$ are defined by the following formula:

10 $$\begin{aligned} \mu_{right} (\theta ) & { = }\left\{ {\begin{array}{*{20}l} {\cos^{2} \theta } \hfill & {\quad {{ - \pi } \mathord{\left/ {\vphantom {{ - \pi } 2}} \right. \kern-0pt} 2} \le \theta \le {\pi \mathord{\left/ {\vphantom {\pi 2}} \right. \kern-0pt} 2}} \hfill \\ 0 \hfill & {\quad {\text{otherwise}}} \hfill \\ \end{array} } \right. \\ \mu_{left} (\theta ) & { = }\left\{ {\begin{array}{*{20}l} {\cos^{2} \theta } \hfill & {\quad - \pi \le \theta \le {{ - \pi } \mathord{\left/ {\vphantom {{ - \pi } 2}} \right. \kern-0pt} 2}, \, {\pi \mathord{\left/ {\vphantom {\pi 2}} \right. \kern-0pt} 2} \le \theta \le \pi } \hfill \\ 0 \hfill & {\quad {\text{otherwise}}} \hfill \\ \end{array} } \right. \\ \mu_{above} (\theta ) & { = }\left\{ {\begin{array}{*{20}l} {\sin^{2} \theta } \hfill & {\quad {0} \le \theta \le \pi } \hfill \\ 0 \hfill & {\quad {\text{otherwise}}} \hfill \\ \end{array} } \right. \\ \mu_{below} (\theta ) & { = }\left\{ {\begin{array}{*{20}l} {\sin^{2} \theta } \hfill & {\quad - \pi \le \theta \le 0} \hfill \\ 0 \hfill & {\quad {\text{otherwise}}} \hfill \\ \end{array} } \right. \\ \end{aligned}$$

## Analysis of basic properties

The histogram of forces^13^ has properties [P1], [P2], [P3] and [P4]. The histogram of position sensing forces and histogram of forces^13^ both compute the gravitational forces between each point of the argument object and each point of the reference object. Based on the histogram of forces, the histogram of position sensing forces introduces the position sensing parameter. The position sensing parameter is computed by the ratio of distance and satisfies properties [P1], [P2] and [P3]. The introduction of the position sensing parameter doesn’t influence properties [P1], [P2]and [P3] of the histogram of forces. So the histogram of position sensing forces also has properties [P1], [P2]and [P3]. The analysis and verification of properties [P1], [P2]and [P3] of the histogram of position sensing forces are completely consistent with the histogram of forces^13^.

The analysis and verification of property [P4] of the histogram of position sensing forces are different with the histogram of forces^13^. The analysis and verification of property [P4] are represented as follows:

Let *I* be a segment of the object *A*, and *J* be a segment of the object *B*. The lengths of segments *I* and* J* are denoted by *x* and *z*. The distance of segments *I* and* J* is denoted by *y*.

Let object *A* be in the direction $\theta$ of the object *B*, the function $f$ is $f(x,y,z)$, the position sensing parameter is $\lambda (\theta ,\nu )$, the function $f_{\theta }^{\nu }$ is $f_{\theta }^{\nu } \left( {x,y,z} \right)$, and the function *F* is $F(\theta ,I,J)$. And if the argument object and reference object exchange, object *B* is in the direction $\theta + \pi$ of object *A*, the function $f$ is $f\left( {z,y,x} \right)$, the position sensing parameter is $\lambda (\theta { + }\pi ,\nu )$, the function $f_{\theta }^{\nu }$ is $f_{\theta + \pi }^{\nu } \left( {z,y,x} \right)$, and the function *F* is $F\left( {\theta + \pi ,J,I} \right)$.

According to Formula (3), for $\left( {x,y,z} \right) \in {\mathbb{R}}_{ + } \times {\mathbb{R}} \times {\mathbb{R}}_{ + }$,$f\left( {z,y,x} \right) = f\left( {x,y,z} \right)$. According to Definition 2 of the position sensing parameter, when the argument object and reference object exchange, the position sensing parameter $\lambda$ which is related to the size and shape of the reference object will change, and $\lambda (\theta ,\nu ) \ne \lambda (\theta { + }\pi ,\nu )$. According to Formula (5), (6), $f_{\theta + \pi }^{\nu } \left( {z,y,x} \right) \ne f_{\theta }^{\nu } \left( {x,y,z} \right)$ and $F\left( {\theta + \pi ,J,I} \right) \ne F(\theta ,I,J)$.

$F(\theta ,I,J)$ and $F\left( {\theta + \pi ,J,I} \right)$ both are greater than 0, there exists $k \in {\mathbb{R}}_{ + }^{*}$,$F\left( {\theta + \pi ,J,I} \right) = k \times F(\theta ,I,J)$.$F\left( {\theta + \pi ,J,I} \right)$ supports the proposition “segment *J* is in the direction $\theta + \pi$ of *I* ”.$F(\theta ,I,J)$ supports the proposition “segment *I* is in the direction $\theta$ of* J* ”. So the histogram of position sensing forces can simultaneously support the proposition “object *A* is in the direction $\theta$ of object *B* ” and proposition “object *B* is in the direction $\theta + \pi$ of object *A* ”. So the histogram of position sensing forces satisfies property [P4].

## Results

In this section, Quad-tree histogram^12^, *F*_0_-histogram^14^, *F*_2_-histogram^14^, and *F*_*p*_-histogram that assess the directional spatial relation are compared by five groups of experiments. *F*_2_-histogram only computes the gravitational forces between each point of the argument object and each point of the reference object and doesn’t compute the spatial position sensing parameters $\lambda$. In other words, if in the formula (5), the position sensing parameter $\lambda (\theta ,\nu )$ is not introduced, the calculation result of formula (7) is *F*_2_-histogram. And if formula (2) is $\varphi (m - n) = 1 \, m > n$, and the formula (5) doesn’t introduce the position sensing parameter $\lambda (\theta ,\nu )$, the calculation result of formula (7) is *F*_0_-histogram.

Quad-tree histogram^12^, *F*_0_-histogram^14^, *F*_2_-histogram^14^, and *F*_*p*_-histogram are noted Q_h_, F_0_, F_2_, and F_p_. For the sake of fairness, these four methods make use of the same handling method of histograms, namely formula (8), (9) and (10).

In five groups of experiments, each degree within the range [0,360) degrees is taken a value to form a serie of angles $\theta$. Quad-tree histogram, *F*_0_-histogram, *F*_2_-histogram and *F*_*p*_-histogram are constructed from this serie of angles $\theta$ including 360 degrees. Q_h_, F_0_, F_2_, and F_p_ respectively provide the degree of truth of the proposition “the argument object A to the right, left, above and below of the reference object B” for five groups of experiments. No matter which direction of the right, left, above and below has the highest degree of truth will be considered the argument object A is in the direction of the reference object B.

Besides, H_c_ which denotes the common intuition of the human is used as the standard for the comparison. To know the common intuition of the human about all scenes in five groups of experiments, statistical analysis about 100 volunteers are done. All volunteers who participate in statistical investigation have nothing to do with this research. The volunteers need to judge which is max between the degree of truth of the proposition “the argument object A to the right of the reference object B”, degree of truth of the proposition “the argument object A to the left of the reference object B”, degree of truth of the proposition “the argument object A to the above of the reference object B” and degree of truth of the proposition “the argument object A to the below of the reference object B”. Then, the number of volunteers selecting the directional spatial relations including ‘right’, ‘left’, ‘above’ and ‘below’ is counted separately. The numbers of H_c_ respectively represent the percentage of volunteers choosing the directions including ‘right’, ‘left’, ‘above’ and ‘below’. The direction most volunteers choose is the common intuition H_c_ of the human.

In five groups of experiments, Q_h_, F_0_, F_2_, and F_p_ respectively provide the degree of truth of the proposition “the argument object A to the right, left, above and below of the reference object B”, the highest degree of truth is bolded, and the common intuition H_c_ of the human is expressed through ‘Max’.

## First experiment

**Figure 6:**
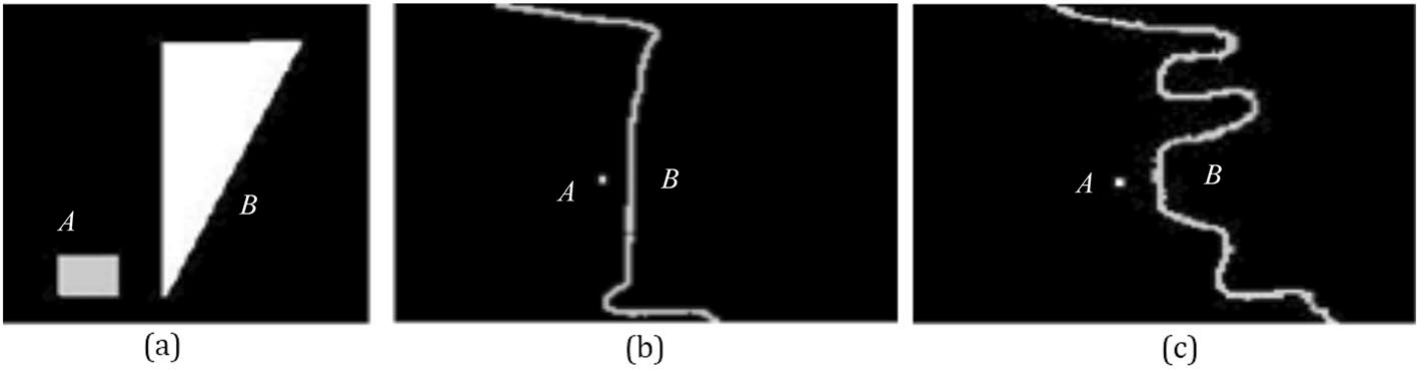
Test images of the first experiment. In images (**a**), (**b**) and (**c**), the small square is the argument object *A*, and other object is the reference object *B*.

In the first experiment, Q_h_, F_0_, F_2_, and F_p_ that assess the directional spatial relation are compared by three scenes of Fig. 6. Results of the first experiment are shown in Table 1.

**Table 1:** Results of the first experiment.

%	(a)					(b)					(c)				
	H_c_	Q_h_	F_0_	F_2_	F_p_	H_c_	Q_h_	F_0_	F_2_	F_p_	H_c_	Q_h_	F_0_	F_2_	F_p_
Right	0	0	0	0	0	0	17	8	15	7	0	5	3	45	50
Left	**Max(90)**	31	38	**59**	**89**	**Max(82)**	27	36	**64**	**68**	**Max(85)**	37	45	**71**	**76**
Above	0	5	4	8	10	2	53	49	34	29	2	43	36	27	22
Below	10	**69**	**62**	41	32	16	**86**	**51**	34	39	13	**66**	**54**	27	26

The highest degrees of truth are in bold.

In Table 1, the common intuition of the human H_c_ represents that the object *A* is at the left of the object *B* in images (a), (b) and (c). F_2_ and F_p_ affirm that *A* is at the left of *B* in images (a), (b) and (c). The above opinions are not agreed with Q_h_ and F_0_, and Q_h_ and F_0_ think that *A* is at the below of *B* in images (a), (b) and (c). Results of F_2_ and F_p_ assessing the directional spatial relation are more rational than results of Q_h_ and F_0_.

## Second experiment

**Figure 7:**
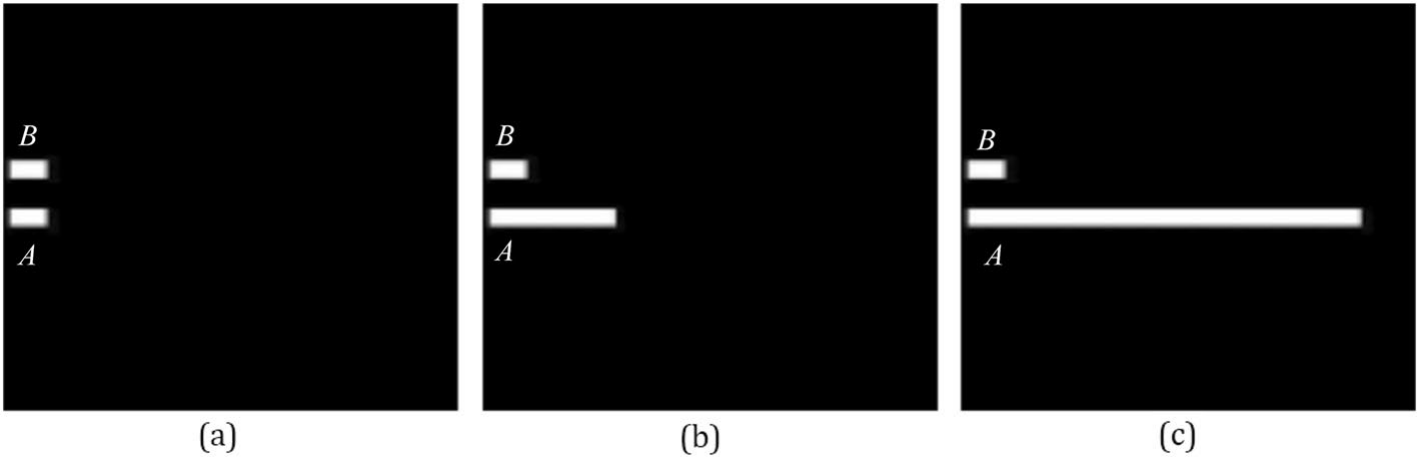
Test images of the second experiment. In images (**a**), (**b**) and (**c**), the square at the bottom is the argument object *A*, and the square at the top is the reference object *B*.

In the second experiment, Q_h_, F_0_, F_2_, and F_p_ that assess the directional spatial relation are compared by three scenes of Fig. 7. Results of the second experiment are shown in Table 2.

**Table 2:** Results of the second experiment.

%	(a)					(b)					(c)				
	H_c_	Q_h_	F_0_	F_2_	F_p_	H_c_	Q_h_	F_0_	F_2_	F_p_	H_c_	Q_h_	F_0_	F_2_	F_p_
Right	0	7	7	7	5	5	45	37	29	35	9	**80**	**63**	34	42
Left	0	7	7	7	5	0	7	6	7	5	0	3	5	7	5
Above	0	0	0	0	0	0	0	0	0	0	0	0	0	0	0
Below	**Max(100)**	**93**	**93**	**93**	**95**	**Max(95)**	**55**	**63**	**71**	**69**	**Max(91)**	21	37	**66**	**69**

The highest degrees of truth are in bold.

In Table 2, the common intuition of the human H_c_ represents that the object *A* is at the below of the object *B* in images (a), (b) and (c). As argument *A* becomes longer, only F_2_ and F_p_ maintain that *A* essentially remains at the below of the object *B* in images (a), (b), and (c), and Q_h_ and F_0_ think that A is at the right of B in image (c). Thus, results of F_2_ and F_p_ assessing the directional spatial relation are more rational than results of Q_h_ and F_0_.

## Third experiment

**Figure 8:**
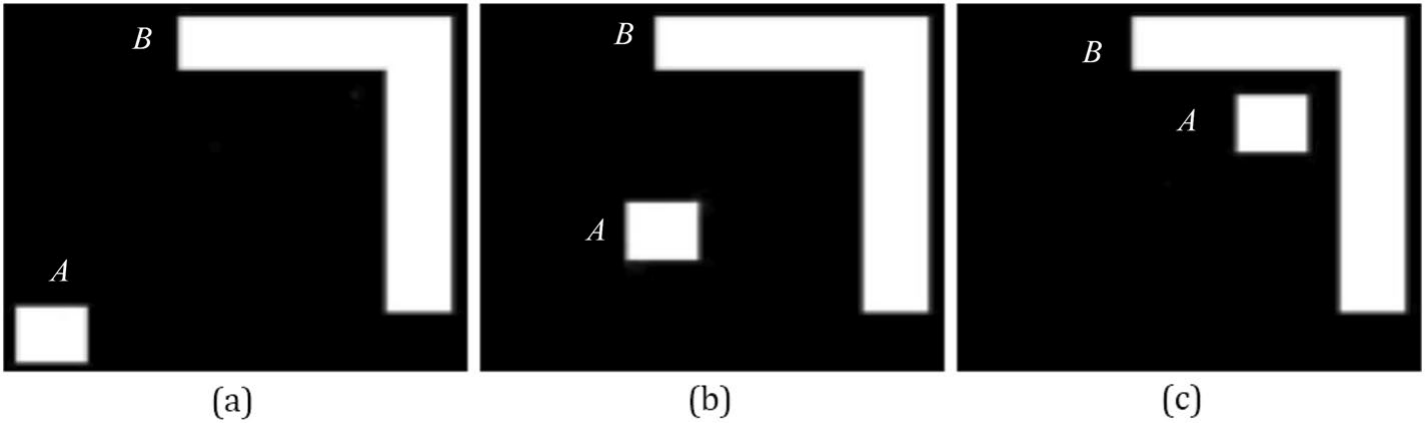
Test images of the third experiment. In images (**a**), (**b**) and (**c**), the small square is the argument object *A*, and other object is the reference object *B*.

In the third experiment, Q_h_, F_0_, F_2_, and F_p_ that assess the directional spatial relation are compared by three scenes of Fig. 8. Results of the third experiment are shown in Table 3.

**Table 3:** Results of the third experiment.

%	(a)					(b)					(c)				
	H_c_	Q_h_	F_0_	F_2_	F_p_	H_c_	Q_h_	F_0_	F_2_	F_p_	H_c_	Q_h_	F_0_	F_2_	F_p_
Right	0	0	0	0	0	0	0	0	0	0	0	32	29	26	30
Left	**Max(88)**	**58**	**60**	**60**	**63**	**Max(83)**	**51**	**51**	**51**	**59**	**Max(80)**	44	49	**50**	**68**
Above	0	0	0	0	0	0	6	6	7	1	2	**51**	43	31	25
Below	12	42	40	40	37	17	49	49	49	40	18	49	**50**	**50**	37

The highest degrees of truth are in bold.

In Table 3, the common intuition of the human H_c_ represents that the object *A* is at the left of the object *B* in images (a), (b) and (c). F_2_ and F_p_ affirm that *A* is at the left of *B* in images (a), (b) and (c). The above opinions are not agreed with Q_h_ and F_0_, Q_h_ thinks that *A* is at the above of *B* and F_0_ think that *A* is at the below of *B* in image (c). Besides, in images (b) and (c), F_2_ thinks that the degrees of truth of ‘Left’ and ‘Below’ are almost equal, and F_p_ think that the degrees of truth of ‘Left’ are max. Thus, results of F_p_ assessing the directional spatial relation are more rational than results of Q_h_, F_0_ and F_2_.

## Fourth experiment

**Figure 9:**
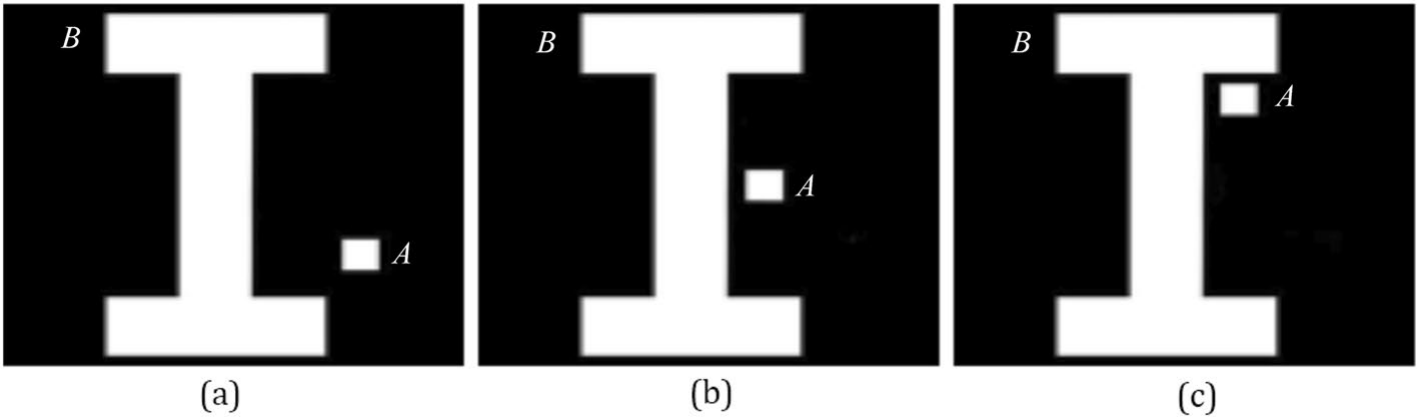
Test images of the fourth experiment. In images (**a**), (**b**) and (**c**), the small square is the argument object *A*, and other object is the reference object *B*.

In the fourth experiment, Q_h_, F_0_, F_2_, and F_p_ that assess the directional spatial relation are compared by three scenes of Fig. 9. Results of the fourth experiment are shown in Table 4.

**Table 4:** Results of the third experiment.

%	(a)					(b)					(c)				
	H_c_	Q_h_	F_0_	F_2_	F_p_	H_c_	Q_h_	F_0_	F_2_	F_p_	H_c_	Q_h_	F_0_	F_2_	F_p_
Right	**Max(97)**	49	**57**	**56**	**78**	**Max(95)**	36	45	**65**	**77**	**Max(91)**	33	45	**50**	**59**
Left	0	0	0	0	0	0	2	2	1	1	0	7	12	17	29
Above	0	28	31	44	21	2	62	54	35	27	8	**74**	**61**	34	42
Below	3	**56**	48	34	27	3	**63**	**55**	35	27	1	38	43	**50**	37

The highest degrees of truth are in bold.

In Table 4, the common intuition of the human H_c_ represents that the object *A* is at the right of the object *B* in images (a), (b) and (c). F_2_ and F_p_ affirm that *A* is at the right of *B* in images (a), (b) and (c). The above opinions are not agreed with Q_h_ and F_0_. Results of F_2_ and F_p_ assessing the directional spatial relation are more rational than results of Q_h_ and F_0_. In image (c), F_2_ thinks that the degrees of truth of ‘Right and ‘Below’ are equal, and *A* is more to the below of *B* than to the above of it. Thus, the opinions of F_2_ are also unrational. According to the above views, results of F_p_ accessing the directional spatial relation are more rational than results of Q_h_, F_0_, and F_2_.

## Fifth experiment

**Figure 10:**
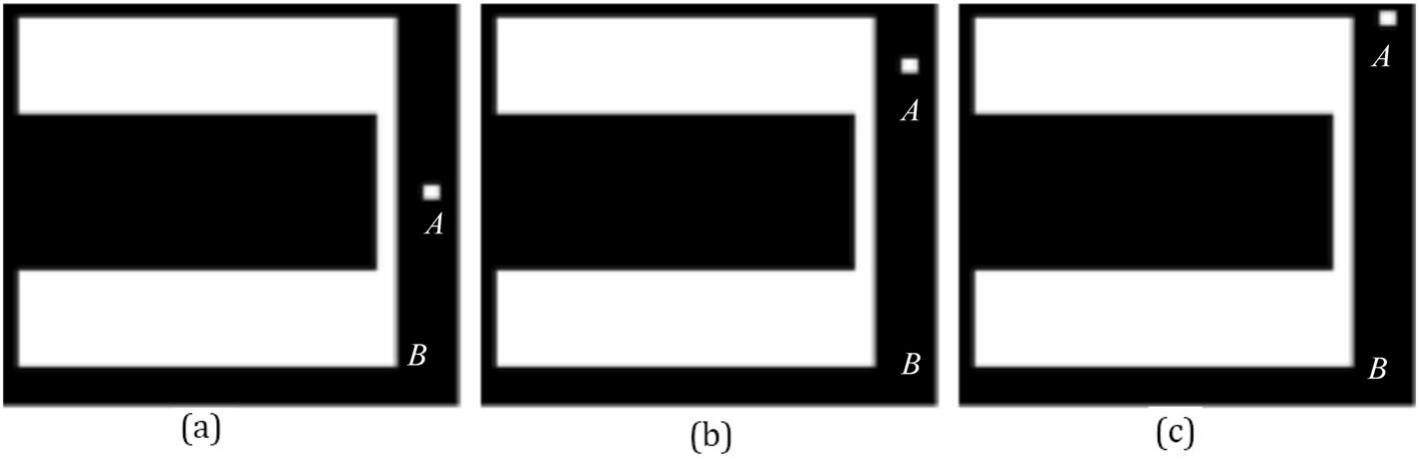
Test images of the fifth experiment. In images (**a**), (**b**) and (**c**), the small square is the argument object *A*, and other object is the reference object *B*.

In the fifth experiment, Q_h_, F_0_, F_2_, and F_p_ that assess the directional spatial relation are compared by three scenes of Fig. 10. Results of the fifth experiment are shown in Table 5.

**Table 5:** Results of the fifth experiment.

%	(a)					(b)					(c)				
	H_c_	Q_h_	F_0_	F_2_	F_p_	H_c_	Q_h_	F_0_	F_2_	F_p_	H_c_	Q_h_	F_0_	F_2_	F_p_
Right	**Max(100)**	**62**	**57**	**62**	**84**	**Max(99)**	**54**	**61**	**73**	**80**	**Max(99)**	**56**	**63**	**71**	**70**
Left	0	0	0	0	0	0	0	0	0	0	0	0	0	0	0
Above	0	36	42	38	16	1	46	40	27	21	1	44	37	29	30
Below	0	36	42	38	16	0	5	8	17	3	0	0	0	0	0

The highest degrees of truth are in bold.

In Table 5, the common intuition of the human H_c_ represents that the object *A* is at the right of the object *B* in images (a), (b) and (c). Q_h_, F_0_, F_2_, and F_p_ express the same opinion with the human intuition H_c_. As argument *A* moves up, only the degree of truth of F_p_ assessing *A* to the right of *B* decreases, the degree of truth of F_p_ assessing *A* to the above of *B* increases, and the degree of truth of F_p_ assessing *A* to the below of *B* decreases. Thus, results of F_p_ accessing the directional spatial relation are more acceptable than results of A_h_, F_0_, and F_2_.

## Discussion

In this paper, the position sensing parameter and histogram of position sensing forces are proposed. The histogram of position sensing forces can simulate the common intuition of the human for the directional spatial relations. It provides a more rational qualitative representation of the directional spatial relationships between two objects of the image. The histogram of position sensing forces is useful to understand the scenes of the image and bridge the gap between the pixels and semantics of the image, and can promote the development of applications such as the linguistic description of spatial relations, object recognition, scene recognition, scene matching, content-based image retrieval, robot navigation, etc.

## Data Availability

The datasets generated during the current study are available from the corresponding author on reasonable request.
